# Determinants of weight, psychological status, food contemplation and lifestyle changes in patients with obesity during the COVID-19 lockdown: a nationwide survey using multiple correspondence analysis

**DOI:** 10.1038/s41366-022-01100-8

**Published:** 2022-03-19

**Authors:** A. Caretto, S. Pintus, M. L. Petroni, A. R. Osella, C. Bonfiglio, S. Morabito, P. Zuliani, A. Sturda, M. Castronuovo, V. Lagattolla, A. Maghetti, E. Lapini, A. M. Bianco, M. Cisternino, N. Cerutti, C. A. Mulas, O. Hassan, N. Cardamone, M. Parillo, L. Sonni, for the ADI (Italian Dietetic and Clinical Nutrition Association) Foundation, E. Urso, C. Bianco, M. Scotto Di Carlo, G. Fantola, M. Vincis, L. Pironi, F. Barbanti, A. Musio, F. Ravaioli, F. Minciullo, R. Balzano, A. R. Cozzolino, I. Castanò, P. Cusano, C. Di Giacomo, M. P. Mollica, Maria Coppola, Daniela Della Rosa, G. Vignola, L. Bolesina, V. Zaccheroni, R. Pullara, G. Caprino, C. Tubili, B. Baccari, G. Monacelli, B. Paolini, B. Martinelli, M. Carella, C. Di Gregorio, D. Cella, W. Facci, M. Lista, S. Giungato, L. Fazzolari, M. Altomare, L. Lo Prinzi, I. Grandone, L. Vigna, P. Di Berardino, L. Messeri

**Affiliations:** 1 Endocrinology, Metabolic diseases and Clinical Nutrition, Ospedale Perrino, Brindisi, Italy; 2 Bariatric Surgery Center, ARNAS Brotzu, Cagliari, Italy; 3 Metabolism and Clinical Nutrition Unit, IRCCS Policlinico S. Orsola, Alma Mater University of Bologna, Bologna, Italy; 4 Epidemiology and Statistics, IRCCS Saverio De Bellis, Castellana Grotte, BA Italy; 5 Obesity Day Center, Messina, Italy; 6 Obesity Day Center, Sorrento, NA Italy; 7 Information technology consultant, Brindisi, Italy; 8 Obesity Rehab Unit, Ospedali Privati Forli, Forlì, Italy; 9 U.O.C. Igiene degli Alimenti e della Nutrizione ASP, Potenza, Italy; 10 Clinical Nutrition, IRCCS “S. De Bellis”, Castellana Grotte, BA Italy; 11 UOSD Medicina Generale a Indirizzo Dietologico ASST, Pavia, Italy; 12 Clinical Dietary Service “Holy Spirit” Hospital Casale Monferrato ASLAL, Alessandria, Italy; 13 UOSD Diabetology, San Camillo Hospital –, Roma, Italy; 14 A.S.P., Catanzaro, Italy; 15 Azienda ospedaliera S. Anna S. Sebastiano, Caserta, Italy; 16 SIAN ASL RM2, Roma, Italy; 17 Endocrinology, Metabolic Diseases and Clinical Nutrition, Ospedale Perrino, Brindisi, Italy; 18 Psychology Unit, Ospedale Perrino, Brindisi, Italy; 19 Bariatric Surgery Center, ARNAS Brotzu, Cagliari, Italy; 20 Metabolism and Clinical Nutrition, IRCCS Policlinico S. Orsola, Alma Mater University of Bologna, Bologna, Italy; 21 Obesity Day Center Valdemone Messina, Messina, Italy; 22 Obesity Day Center, Sorrento, NA Italy; 23 University of Naples Federico II, Napoli, Italy; 24 S.I.A.N. ASP Potenza, Potenza, Italy; 25 UOSD Medicina Generale a indirizzo dietologico, ASST di Pavia, Pavia, Italy; 26 Obesity Rehab Unit, Ospedali Privati Forli, Forlì, Italy; 27 Clinical Dietary Service “Holy Spirit” Hospital Casale Monferrato ASLAL, Alessandria, Italy; 28 UOSD Diabetology, San Camillo Hospital, Roma, Italy; 29 SIAN, ASL RM2 Roma, Italy; 30 Center of Human Nutrition Study, Gubbio, Italy; 31 UOSA Dietetic and Clinical Nutrition, Azienda Ospedaliera-Universitaria “Santa Maria alle Scotte”, Siena, Italy; 32 Obesity Therapy Clinic, S.C. Internal Medicine Hospital, San Severo, FG Italy; 33 Dietetic and Clinical Nutrition Unit, SS Annunziata Hospital, Taranto, Italy; 34 SSD Endocrinology and Dietetic Unit, Galliera Hospital –, Genova, Italy; 35 Digestive Endoscopy, San Pio di Pietralcina Hospital Castellaneta (TA), Castellaneta, Italy; 36 UOC Diabetology, Sandro Pertini Hospital, Roma, Italy; 37 Obesity Day Center, Sant’Agata di Militello (ME), Sicily, Italy; 38 SC Diabetology Dietetic and Clinical Nutrition, Santa Maria Hospital, Terni, Italy; 39 Obesity Center, IRCCS Ca’ Granda Ospedale Maggiore Policlinico, Milano, Italy; 40 Centro Riabilitazione Nutrizionale Villa Pini d’Abruzzo, Chieti, Italy; 41 SIAN ASL Roma 3, Roma, Italy

**Keywords:** Public health, Risk factors, Weight management

## Abstract

**Introduction:**

The corona virus disease 2019 (COVID-19) pandemic forced most of the Italian population into lockdown from 11 March to 18 May 2020. A nationwide survey of Italian Clinical Nutrition and Dietetic Services (Obesity Centers or OCs) was carried out to assess the impact of lockdown restrictions on the physical and mental wellbeing of patients with obesity (PWO) who had follow-up appointments postponed due to lockdown restrictions and to compare determinants of weight gain before and after the pandemic.

**Methods:**

We designed a structured 77-item questionnaire covering employment status, diet, physical activity and psychological aspects, that was disseminated through follow-up calls and online between 2 May and 25 June 2020. Data were analyzed by multiple correspondence analysis (MCA) and multiple linear regression.

**Results:**

A total of 1,232 PWO from 26 OCs completed the questionnaires (72% female, mean age 50.2 ± 14.2 years; mean BMI 34.7 ± 7.6 kg/m^2^; 41% obesity class II to III). During the lockdown, 48.8% gained, 27.1% lost, while the remainder (24.1%) maintained their weight. The mean weight change was +2.3 ± 4.8 kg (in weight gainers: +4.0 ± 2.4 kg; +4.2% ± 5.4%). Approximately 37% of participants experienced increased emotional difficulties, mostly fear and dissatisfaction. Sixty-one percent reduced their physical activity (PA) and 55% experienced a change in sleep quality/quantity.

The lack of online contact (37.5%) with the OC during lockdown strongly correlated with weight gain (*p* < 0.001). Using MCA, two main clusters were identified: those with unchanged or even improved lifestyles during lockdown (Cluster 1) and those with worse lifestyles during the same time (Cluster 2). The latter includes unemployed people experiencing depression, boredom, dissatisfaction and increased food contemplation and weight gain. Within Cluster 2, homemakers reported gaining weight and experiencing anger due to home confinement.

**Conclusions:**

Among Italian PWO, work status, emotional dysregulation, and lack of online communication with OCs were determinants of weight gain during the lockdown period.

## Introduction

For the first time in the past century, the world is experiencing a new pandemic. Italy is one of the most affected countries worldwide in terms of cases per million inhabitants and the mortality rate [1]. The Italian government acted in response to the pandemic by shutting down all nonessential activities and forced a lockdown from 11 March to 18 May 2020. This resulted in the closure of schools, cafes, restaurants and shops, public parks and offices, with a requirement to work from home, and a ban on leaving one’s home unless necessary, i.e., to visit supermarkets and pharmacies and to go to a job in cases in which in-person work was essential (pharmacies, health care facilities, and supermarkets) [2]. The lockdown was then progressively removed, beginning on 12 June [3] (partial reopening of schools and restaurants and cafes could remain open until 6 p.m.). The public health emergency due to COVID-19 caused a cascade of adverse consequences on the entire national health system. Outpatient clinical appointments were postponed, and this had a detrimental impact the management of chronic diseases.

Telemedicine has been used since the beginning of the lockdown to overcome the forced interruption of non-urgent outpatient visits, which were usually scheduled every 3 or 6 months, depending on the condition, and to monitor patients with chronic diseases, such as obesity [4, 5]. Obesity has been shown to be associated with a higher severity of COVID-19 and a worse prognosis. This means a higher hospitalization rate and in-hospital mortality and an increased need for intensive care and invasive mechanical ventilation therapy [6–8].

In addition, measures to control COVID-19 transmission (e.g., lockdown restrictions, social distancing, shutting down indoor activities, working at home) are currently resulting in widespread lifestyle changes (e.g., spending more time at home, reducing PA). Psychological distress from social isolation is exacerbated by forced furlough, layoff, and loss of income [9]. Acute stress and depressive moods have been associated with increased food intake, leading to excess weight gain in persons with obesity (PWO) during lockdown [10], which has also been described in countries other than Italy under similar restrictions [11].

Insecurity about future conditions leading to the purchase of large quantities of foodstuffs, coupled with increased exposure to food at home, reduced physical exercise, heightened stress, fear and emotional eating, may put weight control attempts in jeopardy, particularly for PWO [12, 13]. To the best of our knowledge, the impact of lockdown measures on the mental and physical health of PWO and their weight control has yet to be determined.

In Italy, obesity management is offered under regional health services at three levels of care: (1) primary care; (2) specialist obesity centers (OCs) (mainly outpatient services); and (3) bariatric surgery and inpatient obesity rehabilitation. Clinical Nutrition and Dietetic Services represent the majority of second-level centers for obesity management, and PWO can access them via referral from their general practitioners. Fifty-six clinical nutrition and dietetic services take part in the Italian Association of Dietetics and Clinical Nutrition (ADI) network.

We hypothesized that the COVID-19 pandemic lockdown might have negatively affected the weight control of Italian PWO that were followed up at OCs within the ADI network. In this study, we aimed to do the following:

1. Identify potential changes in weight and weight-related lifestyles among PWO from outpatient OCs in Italy during the COVID-19-related lockdown period (11 March to 18 May 2020).
2. Assess the mental and physical wellbeing of these PWO during lockdown in comparison with their pre-pandemic conditions.
3. Identify determinants of potential weight changes, lifestyle modifications and psychological distress observed among PWO.
4. Assess the potential impact on PWO to maintain communication with clinicians at the OCs during the lockdown using telemedicine to monitor weight, lifestyle and wellbeing.

## Methods

We conducted a multicenter prospective survey targeting PWO previously scheduled for direct examination and then postponed due to lockdown restrictions. All OCs within the ADI network were invited to distribute the survey to patients being followed up by their centers.

Details on each OC are reported at the end of the manuscript in the Study Network Members section.

The Ethics Committee of the Local Health Authority of Brindisi, the coordinating institution for all those participating, approved the study protocol. Patients provided their informed consent to participate before completing the survey.

The survey was carried out via a structured online questionnaire administered by health care personnel during phone calls (for those who wanted a phone consult or who were unfamiliar with the internet), or patients were invited to complete the questionnaire online at a later stage. Those who did not answer were contacted a second time.

The survey took place between 2 May and 25 June, 2020. The dissemination period was prolonged for two weeks after the end of the lockdown to allow patients to complete the questionnaires remotely/online.

### Questionnaire

A structured questionnaire with 77 items was designed to understand the impact of the lockdown restrictions on the weight, lifestyle and health of PWO. The questionnaire was developed *de novo* as a joint effort of the Scientific Committee of the ADI Foundation, including physicians, dieticians, psychologists and data scientists, to provide a multidimensional framework for the survey. The preliminary version of the questionnaire was pilot tested by health personnel from five centers (Brindisi, Cagliari, Bologna, Messina, Sorrento), and the suggestions provided were implemented in the final version. The English and Italian versions of the questionnaire have been included as supplementary material. All variables except for age, height and weight were structured as categorical.

The participants were instructed to check their weight in the morning with light clothes on. Since health personnel routinely accessed patients’ files before calling them, PWO were asked to refer to weight measured at their last visit to the clinic before the lockdown for comparison. In the case of weight changes in the period between the last visit and the beginning of the lockdown, the last self-reported weight before the lockdown was used for comparison. Patients were categorized as overweight (BMI between 24.9 and 29.9 kg/m^2^), obesity class I (BMI between 30.0 and 34.0 kg/m^2^), obesity class II (BMI between 35.0 and 39.9 kg/m^2^), and obesity class III (BMI more than 39.9 kg/m^2^).

The questionnaire was structured as follows:

- Section 1 – Respondents were asked to fill in their sex, age, anthropometric data (self-reported weight and height), education level and current employment status.
- Section 2 – Respondents were asked to report any changes in work activity during the lockdown period (e.g., no change, working from home, work suspension, layoff), changes in daily habits, lifestyle, psychological state, emotional difficulties, sleep quality, body dissatisfaction and psychophysical wellbeing.
- Section 3 – This section focused on food intake and PA. In particular, respondents were asked to report any feelings of hunger, food contemplation (i.e., time spent thinking about food), importance attributed to food, dietary changes, cooking habits, dietary pattern, PA, body weight, and adherence to diet therapy.
- Section 4 – This section assessed the use of different telemedicine procedures (e.g., phone calls, video consultations, e-mail, social media) of respondents with their center/health care consultant, the importance attributed to obesity as a disease, and the perceived need for pharmacological treatment for obesity during lockdown.

Data on dietary changes, cooking habits, and dietary patterns were not included in the current analysis since they were outside the scope of the present paper.

### Statistical analysis

Data are described as the mean (± standard deviation, SD), frequency (%) or median (range) and were compared by using ANOVA or the chi-squared test as appropriate.

The following variables from the questionnaire were analyzed in this study: Section 1 (Demographics, COVID-19 Region, Education, Work and Work type, Anthropometrics), 2 (Work Changes during lockdown, Emotional Difficulties, Sleep modification, Psycho-social wellbeing, Body Dissatisfaction), 3 (Changes in PA, Being on Diet therapy and adherence) and 4 (Willingness to use obesity drugs, Remain in Contact with the OC).

We performed MCA, which is an exploratory multivariate factor analysis, to reduce the dimensionality of large categorical data.

The aim of the MCA was to reduce a set of possibly correlated variables (including demographic and social variables plus categorical answers) into a smaller group of linearly uncorrelated variables (i.e., dimensions). We set at the number of dimensions at two to capture approximately 80% of the variance [14] and to provide a two-dimensional graphical representation. The position of the full set of categories for each variable being investigated (i.e., category-points) in the MCA graph reveals relationships across different variables. For instance, variable categories with a similar profile tend to be grouped together, whereas those that are negatively correlated are positioned on opposite sides of the graph. We performed MCA including weight changes (i.e., weight before and after lockdown), working at home, home confinement, anxiety level, job status during lockdown, emotional difficulties and type of emotional difficulties as variables. Emphasis was placed on socioeconomic characteristics rather than on education since socioeconomic characteristics were more likely to be disparately affected by the stay-at-home orders. MCA allowed us to further analyze work conditions and employment status in relation to psychological trends and the ability to cope with distress and maintain a healthy lifestyle.

To further investigate the relationship between weight behavior during lockdown and potential determinants of weight changes, we performed a multiple linear regression analysis. The dependent variable was built as the difference between weight after the lockdown and before. Consequently, the coefficient with a negative sign (-β) indicates a pre-restriction weight greater than the postrestriction weight. All independent variables were categorical. There were two criteria to choose those variables to be used for the regression. The first was the substantive contribution to each aspect of weight change, and the second was the contribution of each variable to the total variance explained by the model. To address collinearity, we used the variance inflation factor metric, which measures the correlation and strength of correlation between the explanatory variables in a regression model. The variance inflation factor for each of the explanatory variables in the model and the overall mean value were less than 2. This indicates that multicollinearity was not an issue.

Italian regions were subgrouped according to the cumulative standardized daily incidence of COVID-19 infection/100,000 inhabitants at the end of June 2020. A “high” incidence area was arbitrarily defined as >1,000 cases/100,000 inhabitants, “medium” incidence was defined as 200 to 999 cases/100,000 inhabitants, and “low” incidence was defined as < 200 cases/100,000 inhabitants.

## Results

### Survey responses

Twenty-six centers (46% of those invited) agreed to recruit participants. Details on each center are reported at the end of the manuscript in the Study Network Members section. The total number of phone contacts was 2319. Of these, 1087 patients (46%) did not complete the questionnaire for time constraints or privacy reasons. Out of the 1,232 patients who participated in the survey, 875 respondents (71%) were questioned during a phone call, whereas 357 (29%) completed the online questionnaire. No differences in baseline characteristics and responses were observed between the two groups. The median number of patients enrolled in each center was 48 (range 4–160).

### Demographics and anthropometrics of respondents

The mean age of the respondents was G50.5 ± 14.3 years (range 11–85). More than 50% of participants lived in southern Italy, whereas approximately 30% lived in northern Italy. Seventy-two respondents were female, and the proportion was consistent across all regions. Respondents had finished middle school (29%) or high school (41%) and were employed (37%), homemakers (16%) or retired (15%). The mean weight was 94.9 kg (range 57-200) at the time of interview, with a mean BMI of 34.5 ± 7.49 kg/m^2^ (range 21.8–58.6). BMI categories were overweight 24%, obesity class I 30%, obesity class II 22%, and obesity class III 18%.

### Lifestyle changes during lockdown

During the lockdown period, 48.8% (*n* = 601) of PWO experienced weight gain and 27.1% (*n* = 334) weight loss, while body weight remained unchanged in 24.1% (*n* = 297) (Table 1). Mean weight change was +2.3 (±4.8 kg). Weight gain was 4.2 kg (±2.6 kg) among PWO who gained weight, with a percentage increase of 4.7 ± 2.9%. Differences in weight gain between single job categories did not reach statistical significance.

**Table 1:** General characteristics of participants by weight change. ADI STUDY. May–June. 2020. Italy.

Variable	Whole sample	Weight change			*P* value*
		Unchanged	Weight loss	Weight gain	
*N* (1232)		297 (24.1%)	334 (27.1%)	601 (48.8%)	
Age (Years)	50.48 (14.26)	52.15 (13.81)	48.14 (15.24)	50.89 (13.75)	0.001
Sex					
Female	913 (72.46%)	214 (24.0%)	239 (26.8%)	438 (49.2%)	0.91
Male	347 (27.54%)	82 (24.5%)	93 (27.8%)	160 (47.8%)	
Weight (kg)	93.10 (22.74)	92.33 (25.33)	90.31 (21.06)	95.01 (22.45)	<0.001
Weight classes (kg)					
<60	27 (2.16%)	10 (37.0%)	11 (40.7%)	6 (22.2%)	<0.011
60–70	129 (10.32%)	39 (30.7%)	39 (30.7%)	49 (38.6%)	
71–80	251 (20.08%)	56 (22.8%)	69 (28.0%)	121 (49.2%)	
81–90	227 (18.16%)	60 (27.1%)	65 (29.4%)	96 (43.4%)	
91–100	249 (19.92%)	42 (17.5%)	68 (28.3%)	130 (54.2%)	
101–120	241 (19.28%g)	52 (22.2%)	60 (25.6%)	122 (52.1%)	
121–150	86 (6.88%)	24 (28.2%)	14 (16.5%)	47 (55.3%)	
>150	40 (3.20%)	10 (29.4%)	7 (20.6%)	17 (50.0%)	
Body mass index (Kg/m2)	34.51 (7.49)	34.10 (8.01)	33.26 (7.02)	35.36 (7.44)	<0.001
Body mass index status					
Normal weight	68 (5.40%)	24 (35.8%)	23 (34.3%)	20 (29.9%)	<0.001
Overweight	299 (23.73%)	74 (25.2%)	93 (31.6%)	127 (43.2%)	
Obesity Class I	381 (30.24%)	82 (22.0%)	102 (27.4%)	188 (50.5%)	
Obesity Class II	281 (22.30%)	64 (23.8%)	74 (27.5%)	131 (48.7%)	
Obesity Class III	231 (18.33%)	52 (22.9%)	42 (18.5%)	133 (58.6%)	
Work status					
Unemployed	111 (8.82%)	25 (22.9%)	37 (33.9%)	47 (43.1%)	0.66
Homemaker	208 (16.52%)	45 (22.3%)	48 (23.8%)	109 (54.0%)	
Artisan/Trader/Farmer	82 (6.51%)	19 (23.8%)	24 (30.0%)	37 (46.3%)	
Public employee	207 (16.44%)	54 (26.7%)	55 (27.2%)	93 (46.0%)	
Private employee	251 (19.94%)	53 (21.5%)	74 (30.1%)	119 (48.4%)	
Self-Employed	104 (8.26%)	23 (22.5%)	22 (21.6%)	57 (55.9%)	
Retired	191 (15.17%)	51 (27.6%)	48 (25.9%)	86 (46.5%)	
Other	105 (8.34%)	24 (24.0%)	25 (25.0%)	51 (51.0%)	
Working from home					
No	820 (73.67%)	202 (25.2%)	209 (26.1%)	390 (48.7%)	0.30
Yes	293 (26.33%)	59 (20.7%)	81 (28.4%)	145 (50.9%)	
Covid-19 region					
Area with low incidence	299 (23.56%)	55 (19.0%)	88 (30.4%)	146 (50.5%)	0.14
Area with medium incidence	332 (26.16%)	87 (26.9%)	88 (27.2%)	149 (46.0%)	
Area with high incidence	638 (50.28%)	155 (25.0%)	158 (25.5%)	306 (49.4%)	
Education					
Primary school	103 (8.19%)	28 (27.5%)	28 (27.5%)	46 (45.1%)	0.46
Middle school	372 (29.57%)	91 (24.9%)	88 (24.1%)	186 (51.0%)	
High school	525 (41.73%	117 (22.9%)	155 (30.3%)	239 (46.8%)	
Graduation	258 (20.51%)	60 (24.0%)	63 (25.2%)	127 (50.8%)	

Cell values represent mean (SD) or frequency (%) as appropriate.

Table 1 summarizes the results by subgrouping participants according to weight change status (unchanged, lost and gained). No statistically significant differences were found among the three groups of patients for age, sex, level of education, work status, working from home or not, and area of residence based on the incidence (low-medium-high) of positive COVID cases. The lifestyle and emotional characteristics of the participants by weight change are shown in Table 2. Approximately 37% of all respondents had increased emotional difficulties, mostly fear and dissatisfaction, while boredom and depression were less frequent. Sixty-one percent of all PWO reduced their PA, and approximately 55% of participants who experienced a change in sleep quality/quantity with insomnia or early awakening were weight gainers (*p* > 0.001).

**Table 2:** Lifestyle and emotional characteristics of participants by weight change. ADI STUDY, May–June, 2020, Italy.

Variable		Weight change			*p* value*
	whole sample	Unchanged	Weight loss	Weight gain	
	*N* (%)	*N* (%)	*N* (%)	*N* (%)	
*N*	1232 (100%)	297 (24.1%)	334 (27.1%)	601 (48.8%)	
Emotional difficulties					
Unchanged	714 (58.24%)	187 (26.8%)	217 (31.1%)	294 (42.1%)	<0.001
Increased	452 (36.87%)	84 (18.8%)	85 (19.1%)	277 (62.1%)	
Decreased	60 (4.89%)	17 (30.9%)	21 (38.2%)	17 (30.9%)	
Sleep modification					
No	558 (44.60%)	137 (25.2%)	177 (32.5%)	230 (42.3%)	<0.001
Yes	693 (55.40%)	157 (23.2%)	152 (22.5%)	368 (54.4%)	
Psychophysical wellbeing					
Unchanged	626 (50.48%)	180 (29.7%)	181 (29.8%)	246 (40.5%)	<0.001
Increased	205 (16.53%)	43 (21.5%)	96 (48.0%)	61 (30.5%)	
Decreased	409 (32.98%)	69 (17.1%)	54 (13.4%)	281 (69.6%)	
Body dissatisfaction					
No	250 (19.90%)	68 (28.0%)	101 (41.6%)	74 (30.5%)	<0.001
Yes	1006 (80.10)	227 (23.1%)	231 (23.5%)	523 (53.3%)	
Changes in physical activity (PA)					
PA reduction	739 (60.87%)	160 (21.7%)	165 (22.3%)	414 (56.0%)	<0.001
PA carried out at home	208 (16.99%)	57 (27.4%)	90 (43.3%)	61 (29.3%)	
PA increased	74 (6.13%)	22 (29.7%)	40 (54.1%)	12 (16.2%)	
No PA	194 (16.01%)	52 (26.8%)	37 (19.1%)	105 (54.1%)	
Being on a diet therapy before lockdown					
No	379 (30.69%)	111 (29.8%)	41 (11.0%)	220 (59.1%)	<0.001
Yes	856 (69.31%)	179 (21.1%)	291 (34.3%)	378 (44.6%)	
Difficulty in following a diet					
No	493 (48.81%)	148 (30.3%)	239 (48.9%)	102 (20.9%)	<0.001
Yes	517 (51.19%)	81 (15.8%)	81 (15.8%)	350 (68.4%)	
Willingness to use obesity drug					
No	1168 (96.05%)	239 (24.3%)	304 (30.9%)	441 (44.8%)	<0.001
Yes	48 (3.95%)	38 (21.8%)	17 (9.8%)	119 (68.4%)	
Remained in contact with your obesity center					
No	297 (27.17%)	83 (28.2%)	41 (13.9%)	170 (57.8%)	<0.001
Yes	683 (62.49%)	140 (20.7%)	274 (40.5%)	263 (38.8%)	
No referral center	113 (10.34%)	28 (25.0%)	4 (3.6%)	80 (71.4%)	

Cell values represent frequencies (%).

### Correlations with weight gain

Table 2 lists significant correlations between the investigated variables and weight gain (*p* < 0.001). Psychophysical wellbeing was decreased in 69.6% of PWO with weight gain and in 17% and 13% of obese patients with unchanged weight or weight loss, respectively. Emotional difficulties (weight gain 62%, unchanged 17% and weight loss 21%), changes in sleep quality/quantity (54%, 23%, and 22%), reduced PA (56%, 21%, 22%) and difficulty following the diet (68%, 15%, and 15%) were more prevalent in those reporting weight gain.

### Use of telemedicine

The majority (62.5%) of PWO had the opportunity to keep in touch with their health care consultant via telephone, social media, video calls or emails. Lack of contact was found to significantly correlate with weight gain (weight gainers 57.8%, stable weight 28.2%, and weight loss 13.9%) (*p* value < 0.001).

Fifteen percent of participants considered the use of obesity medications. This statement was made by 68.4% of those who gained weight, 21.8% of those with stable weight and 9.8% of PWO experiencing weight loss (*p* value < 0.001).

### Multiple linear regression analysis

The effect of some determinants on weight difference (weight after lockdown vs. weight before lockdown) was also probed. Table 3 shows that compared to participants with unchanged psychophysical wellbeing, those reporting increased psychophysical wellbeing experienced a statistically significant average decrease in weight of 2.27 kg (*p* < 0.001), while those with decreased psychophysical wellbeing gained 1.02 kg (*p* = 0.001). Compared to participants who self-identified as physically inactive, those who reported an increase in PA lost 1.94 kg on average. Respondents who reported “increased food contemplation” compared to those who did not report changes had a coefficient of +1.81 (*p* < 0.001) (an average weight gain of 1.81 kg), while participants with “decreased food contemplation” had a statistically significant weight change of −1.87 kg (*p* < 0.001) compared to those who did not report weight change. Respondents who attributed “greater value to food than before” compared to those who attributed “unchanged value to food” had a coefficient of +1.15 (*p* < 0.001). PWO in contact with their OCs had a coefficient of −1.10 (*p* < 0.001). This implies that they had an average weight loss of 1.10 kg compared to the reference group, that is, those who were not in contact with their OCs. Respondents without a referral OC at the time of the survey/study had a coefficient of +1.01 (*p* = 0.005). The interpretation is that they had an average weight gain of 1.01 kg compared to the reference group, i.e., those who had a referral OC but who were not in contact.

**Table 3:** Multiple linear regression. effect of some determinants on weight change. ADI study. May–June. 2020. Italy.

Weight differences	Coeff	95% IC
Psychophysical wellbeing		
Unchanged	0.00	
Increased	−2.25**	−3.05; −1.46
Decreased	1.19**	0.54; 1.83
Thought of food		
Unchanged	0.00	
Decreased	−1.47**	−2.42; −0.53
Increased	1.43**	0.72; 2.14
Physical Activity Changes (PA)		
PA reduction	0.00	
PA carried out at home	−1.27**	−1.95; −0.58
PA increased	−2.29**	0.07; 1.77
Value you give to food		
Unchanged	0	
Decreased	−0.07	−1.52; 1.39
Increased	0.92*	0.07; 1.77
Remained in contact with your obesity center		
No	0.00	
Yes	−0.98*	−1.61; −0.35
I don’t have a referral center	1.21*	0.19; 2.23
Body dissatisfaction		
No	0.00	
Yes	0.96*	0.27; 1.64

Age and Sex adjusted estimates. In the regression model. the variable under study is the weight difference (Weight after restrictions- Weight before restrictions). Consequently the coeff. with a negative sign (-β) (Weight t1 < Weight t0) indicates a pre-restriction weight greater than the post weight.

*p value < 0.05; **p value < 0.001.

Age and sex were not associated with weight change.

### Multiple correspondence analysis

As a previous step, we performed a simple correspondence analysis to probe the association between variables. Each single variable evidenced a strong statistically significant association with each other (*p* < 0.001). We choose only two dimensions, that is, two axes from a Cartesian plane, as they explained the highest percentage of variability.

**Fig. 1:**
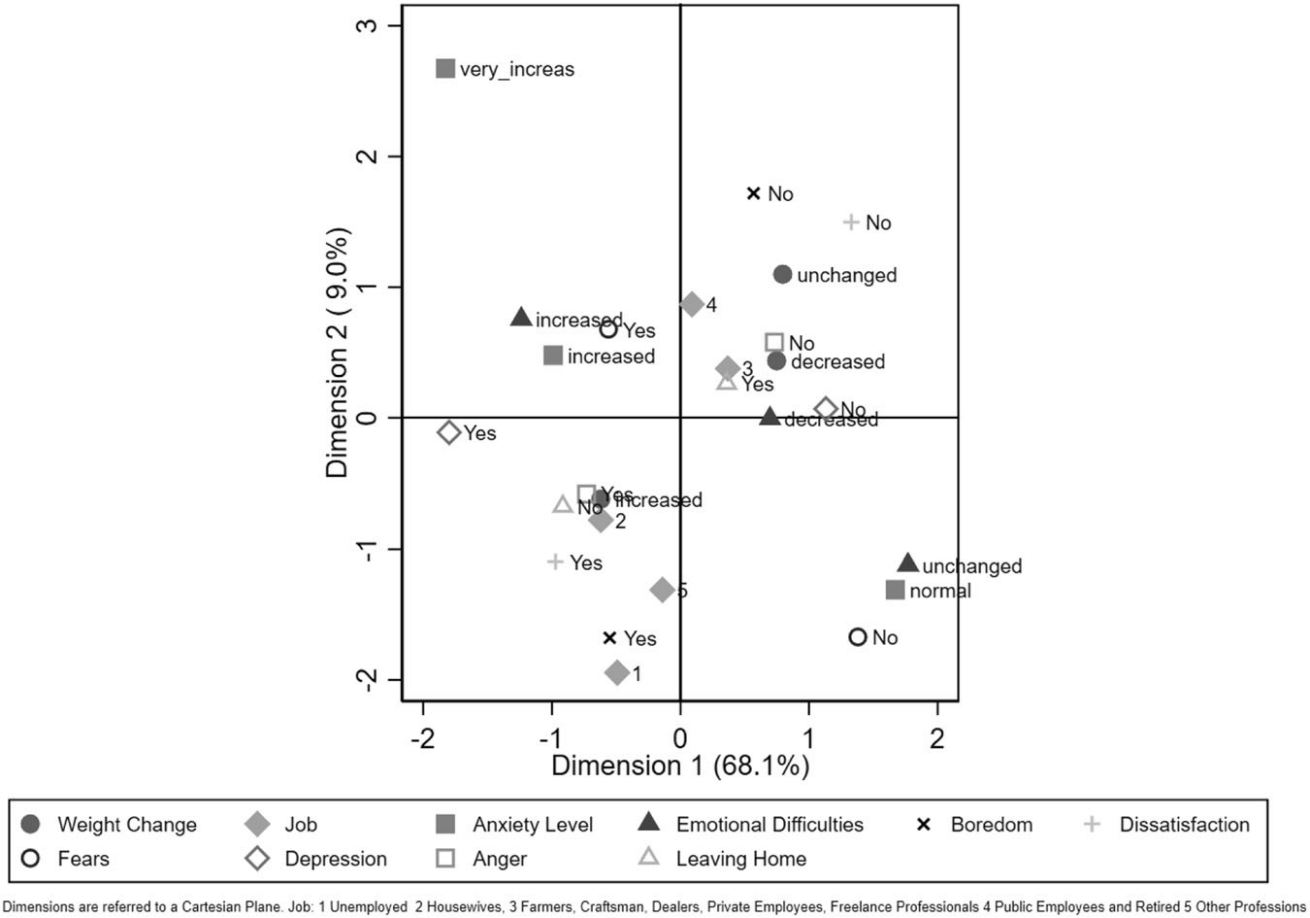
Multiple correspondence analysis. Weight changes and emotional characteristics.

The results from MCA are visually shown in Fig. 1. In addition to all the variables that express the moods related to emotional problems, such as anxiety, boredom, and fear, we also included the variables linked to weight change and the variable “work”. Cluster 1 shows a lifestyle prior to the COVID-19 lockdown that has improved or not changed, and Cluster 2 shows a worsened lifestyle.

Cluster 1 (unchanged or improved lifestyle) is linked to job categories 3 and 4 (artisans/traders/farmers, private employees, self-employed; public employees, retired people). These individuals seem to have coped well with the emotional problems related to the lockdown: unchanged or decreased weight, no anger, no depression, no boredom, no dissatisfaction, normal anxiety level, and unchanged emotional difficulties.

Cluster 2 is linked to a different employment status than those cited for Cluster 1. The majority of unemployed people and those working from home were in this cluster, and they reported experiencing depression, boredom, dissatisfaction, weight gain, and worsened lifestyle. Included in this cluster were individuals who could not leave home, those who experienced greater food contemplation, those who ate more than before and those who wanted help from obesity medication. It should be noted that homemakers seem to form a cluster of their own: they gained weight, felt angry, and confined themselves at home.

## Discussion

To our knowledge, this is the first study that estimated large-scale social and demographic determinants of weight gain and linked them to psychological and lifestyle-related variables during the COVID-19 lockdown among PWO. The main finding was that age and sex did not affect weight gain, while work status did. Homemakers, those who worked at home and people laid off from their jobs experienced greater weight gain than respondents who were retired or worked outside of the home.

The results from 1232 PWO showed that the lockdown restrictions had a significant negative impact on body weight and emotional and psychophysical wellbeing. These measures resulted in a reduction in PA in the majority of responders. Increased food consumption and food intake and difficulties in compliance with diet therapy were also been reported. Poor lifestyle was correlated with other variables, such as type of work, home confinement, and not keeping in contact with one’s center/health care consultant.

In the present survey, almost half of respondents gained weight during lockdown. Nevertheless, a surprisingly high percentage was able to maintain or even reduce their weight. This range of responses can be attributed to differences in the energy balance between food consumption and PA.

What influenced these differences? We found that over one-third of all respondents declared increased emotional difficulties, and more than 50% experienced insomnia or reduced sleep. These behaviors may have a negative impact on psychological health and favor weight gain [9, 13]. Body dissatisfaction was also significantly linked to weight gain. This is a well-known factor associated with binge eating and depression [15] and with a higher risk of discontinuation of obesity treatment [16].

In the present study, PWO declaring increased food contemplation and perceived increased importance given to food gained weight. Within this mindset, increased exposure and food availability due to stockpiling of food during stay-at-home orders [17] might trigger overconsumption.

Further insight has been provided by MCA. We were able to identify two main clusters: respondents with unchanged or even improved lifestyles during the COVID-19 lockdown (Cluster 1) and those who had worsened lifestyles in this time period (Cluster 2). We further linked the clusters to social and psychological determinants of weight gain.

Cluster 1 included most patients (57.9%) who were able to maintain or even decrease their body weight during the lockdown. Common working conditions for respondents within Cluster 1 ranged from retirement, private or public employment, artisan/trader/farmer or self-employment. Respondents within this cluster reported no experience of significant emotional difficulties, having more freedom of movement, and having the opportunity to exercise at home. PA and exercise training are associated with reduced cardiovascular risk and improved cardiometabolic risk factors, and they facilitated weight loss by creating a negative energy balance [18].

The present study highlighted the correlation between psychological and lifestyle variables (e.g., emotional difficulties, reduction of PA, a relationship with food in terms of pleasure, intrinsic value, and hunger) and gaining weight. These variables in Cluster 2 again correlated with work status (i.e., being a homemaker, unemployed or working at home). Respondents within Cluster 2 appeared to be more interested in taking weight control drugs if they had the opportunity to do so.

Negative emotional states seem to represent potential pathways toward a worsening lifestyle by increasing body weight and deteriorating physical and mental health [19]. These results are in line with those from a small series of PWO enrolled in a weight loss program [20]. In these patients, weight gain during lockdown was predicted by loneliness and working from home, and their negative impact on emotional eating, PA, and the ability to choose healthy foods. Almandoz et al. [17] reported that PWO experiencing stay-at-home orders reported increased anxiety, depression, stress eating, and more difficulty in achieving weight loss goals. As in the present study, education level did not predispose patients to weight gain.

PWO who were not monitored remotely by their OCs during the lockdown reported increased difficulties in weight control. Our study suggests the utility of telemedicine, which might prove useful especially in PWO with more social fragility and emotional dysregulation leading to increased food intake. These patients might need more frequent periodic check-ups and a targeted approach. Moreover, although we did not test them in the present study, third-generation cognitive-behavioral techniques might prove useful in this respect. For example, mindfulness training delivered remotely seems promising in reducing impulsive and binge eating among adults with overweight and obesity [21].

Over two-thirds of the respondents who gained weight would have considered it appropriate to use obesity medications during the lockdown. This finding contrasts with the very limited use of obesity medications in Italy [22]. Drug therapy for obesity is already considered among the pillars of the clinical management of obesity [23] and will become even more so once novel, more powerful agents become available on the market [24]. Obesity medication may also be suitable and acceptable for short-term weight management in situations, such as during the lockdown, where for a limited amount of time, a patient is intermittently exposed to environmental factors that promote weight gain and lifestyle choices are limited [25]. While lockdowns like this are not likely to become the norm for us in the future, the colder and darker winter months could present a similar scenario for obesity patients in many countries, as individuals may experience emotional distress due to the lack of sunlight or lifestyle changes due to lower availability of fresh food and fewer options to exercise outdoors. Likewise, summers in very hot countries may produce similar effects when individuals exercise less due to the heat.

Attention must also be given to PWO who need but cannot afford obesity medications, such as our unemployed participants or individuals experiencing layoffs in Cluster 2. This issue will require close attention from policy-makers in the future to establish a cost-effective means of providing funding and insurance coverage.

What are the takeaways from this survey for clinical practice? We believe that when outpatient visits are not feasible, virtual visits must be put in place to provide patients with regular access to health care. It is also essential to target those population groups that may need an increased level of telehealth services to equalize opportunities for weight control (during lockdown/winter/heat waves but also after the height of this pandemic to get them back on track). These PWO may need other support in addition to more frequent remote consultations, which could be obesity medication, stress management/mindfulness training or lifestyle coaching.

Future studies could focus on research using a similar methodology on the impact of economic recessions (including those linked to the COVID-19 pandemic) on PWO from countries other than Italy. Additionally, it could be worthwhile to assess the role of body dissatisfaction and bariatric surgery and determinants of weight changes during critical periods.

The present study has several limitations. We believe that the sampling of patients could be a source of bias. Since 46% of contacted patients declined to participate in the survey, we cannot assume that our sample is fully representative of PWO followed up at OCs in Italy during the lockdown. Additionally, since we did not adjust the analysis for the timing/date of interviews, this could have decreased some of the effect sizes observed.

## Conclusions

We were able to identify different clusters of PWO during the COVID-19 lockdown: (1) those who struggled to maintain weight/healthy lifestyle/wellbeing vs. (2) those who reported no changes or improvements in either, and we were able to identify social and psychological factors associated with both clusters.

Our findings focus attention on the follow-up of chronic diseases such as obesity during the COVID-19 pandemic. Political choices that focus on preventing the interruption of follow-up medical care and decreasing the impact on public health should be prioritized; for example, training in telemedicine use could be added to health professionals’ education [26]. Shared strategies are urgently needed to face the double challenges posed by obesity and the COVID-19 pandemic. The duration of the pandemic and the persistence of restrictive policies by the states (not only Italian policies) require answers that are as common as possible and adaptable to all chronic diseases.

## Supplementary information

Survey Questionnaire (English translation)

Original Survey Questionnaire (Italian)

## References

[1] COVID-19 Data Repository by the Center for Systems Science and Engineering (CSSE) at Johns Hopkins University. Baltimore, Maryland, USA. https://github.com/CSSEGISandData/COVID-19.

[2] Decree of the President of the Italian Council of Ministers. Rome, Italy. http://governo.it/sites/new.governo.it/files/dcpm20202322.pdf

[3] Data from the Italian Ministry of Health. Rome, Italy. https://www.istat.it/it/files//2020/12/Rapp_Istat_Iss.pdf

[4] Telemedicine-Opportunities and developments in member states, 2nd ed. Geneva, Switzerland: WHO press; 2010. https://www.who.int/goe/publications/goe_telemedicine_2010.pdf.

[5] Runfola M, Fantola G, Pintus S, Iafrancesco M, Moroni R (2020). Telemedicine implementation on a bariatric outpatient clinic during COVID-19 pandemic in Italy: an unexpected hill-start. Obes Surg.

[6] Farias Costa F, Reis Rosario W, Ribeiro Farias AC, Guimaraes de Souza R, Duarte Gondim CS, Assunçao (2020). Metabolic syndrome and COVID-19: An update on the associated comorbidities and proposed therapies. Diabetes Metab Syndr.

[7] Finer N, Garnett SP, Bruun JM (2020). COVID 19 and obesity. Clin Obes.

[8] Yang J, Tian C, Chen Y, Zhu C, Chi H, Li J (2020). Obesity aggravates COVID-19: an updated systematic review and meta-analysis. J Med Virol.

[9] Salari N, Hosseinian-Far A, Jalali R, Vaisi-Raygani A, Rasoulpoor S, Mohammadi M, et al. Prevalence of stress, anxiety, depression among the general population during the COVID-19 pandemic: a systematic review and meta-analysis. Glob Health. 2020;16:57. 10.1186/s12992-020-00589-w PMC7338126 32631403

[10] Di Renzo L, Gualtieri P, Pivari F, Soldati L, Attinà A, Cinelli G (2020). Eating and lifestyle changes during COVID-19 lockdown: an Italian survey. J Transl Med.

[11] Rodríguez-Pérez C, Molina-Montes E, Verardo V, Artacho R, García-Villanova B, Guerra-Hernández EJ (2020). Changes in dietary behaviours during the COVID-19 outbreak confinement in the Spanish COVIDiet Study. Nutrients.

[12] Popkin BM, Du S, Green WD, Beck MA, Algaith T, Herbst CH (2020). Individuals with obesity and COVID-19: a global perspective on the epidemiology and biological relationships. Obes Rev.

[13] Marchitelli S, Mazza C, Lenzi A, Ricci E, Gnessi L, Roma P (2020). Weight gain in a sample of patients affected by overweight/obesity with and without a psychiatric diagnosis during the covid-19 lockdown. Nutrients.

[14] Le Roux B, Rouanet H. The method of multiple correspondence analysis. In Multiple correspondence analysis. Thousand Oaks, California, USA: SAGE Publications, Inc. 2010. p. 34–67.

[15] Grilo CM, Ivezaj V, Lydecker JA, White MA (2019). Toward an understanding of the distinctiveness of body-image constructs in persons categorized with overweight/obesity, bulimia nervosa, and binge-eating disorder. J Psychosom Res.

[16] Dalle Grave R, Calugi S, Molinari E, Petroni ML, Bondi M, Compare A, Marchesini G, QUOVADIS Study Group. (2005). Weight loss expectations in obese patients and treatment attrition: an observational multicenter study. Obes Res.

[17] Jaime P, Almandoz JP, Xie L, Jeffrey N, Schellinger JN, Mathew NS (2020). Impact of COVID-19 stay-at-home orders on weight-related behaviors among patients with obesity. Clin Obes.

[18] Petridou A, Siopi A, Mougios V (2019). Exercise in the management of obesity. Metabolism.

[19] Torres SJ, Nowson CA (2017). Relationship between stress, eating behavior, and obesity. Medicine 2007; 23: 887-894.adults with overweight and obesity: a systematic review and meta-analysis. Obes Res Clin Pract.

[20] Borgatti AC, Schneider-Worthington CR, Stager LM, Krantz OM, Davis AL, Blevins M (2021). The COVID-19 pandemic and weight management: effective behaviors and pandemic-specific risk factors. Obes Res Clin Pract.

[21] Taylor VA, Moseley I, Sun S, Smith R, Roy A, Ludwig VU (2021). Awareness drives changes in reward value which predict eating behavior change: probing reinforcement learning using experience sampling from mobile mindfulness training for maladaptive eating. J Behav Addict..

[22] Squadrito F, Rottura M, Irrera N, Minutoli L, Bitto A, Barbieri MA (2020). Anti-obesity drug therapy in clinical practice: Evidence of a poor prescriptive attitude. Biomed Pharmacother.

[23] Pharmacotherapy in Obesity Management. Canadian adult obesity clinical practice guidelines. 2020. https://obesitycanada.ca/guidelines/pharmacotherapy/

[24] Finer N (2021). Future directions in obesity pharmacotherapy. Eur J Intern Med.

[25] Apovian CM, Aronne LJ, Bessesen DH, McDonnell ME, Murad MH, Pagotto U (2015). Endocrine Society. Pharmacological management of obesity: an Endocrine Society clinical practice guideline. J Clin Endocrinol Metab.

[26] Haucke E, Walldorf J, Ludwig C, Buhtz C, Stoevesandt D, Clever K. Application of telepresence systems in teaching - transfer of an interprofessional teaching module on digital aided communication into the block training “internal medicine” during the Covid-19 pandemic. GMS J Med Educ. 2020;37:Doc84. 10.3205/zma001377. 10.3205/zma001377 PMC7740017 33364363

