# Supplementary material for: Determinants of weight, psychological status, food contemplation and lifestyle changes in patients with obesity during the COVID-19 lockdown: a nationwide survey using multiple correspondence analysis
Source: Int J Obes (Lond). 2022 Mar 19;46(7):1280–7. doi: 10.1038/s41366-022-01100-8 (PMC8933751; doi:10.1038/s41366-022-01100-8)
Supplement: Supplementary file 1 — Survey Questionnaire (English translation) [file 41366_2022_1100_MOESM1_ESM.docx]

Pag

Fondazione ADI

QUESTIONNAIRE DAY 2020

Your home lifestyle during the COVID-19 pandemic

*The questions have the purpose to understand the impact of restrictions due to Coronavirus pandemic on your lifestyle.*

Date of compilation _________

Obesity Day Center_____________________________ Town ________________________ PR ___

Sex 🔿 M 🔿 F Age ____ Weight _____kg Stature _____cm BMI _______

Profession:

- Unemployed 🔿 Homemaker 🔿 Artisan/trader/farmer 🔿 Public Employee 🔿 Private Employee 🔿 Freelance 🔿 Retired 🔿 Other………..

Scholar level: 🔿 Primary 🔿 Middle school 🔿 High school 🔿 Graduation

Working from home this month: 🔿 Yes 🔿 No

Did you stop work?: 🔿 Yes 🔿 No

-if yes for: 🔿 personal choice 🔿 Due to restrictions 🔿 layoffs

Could you leave home during restrictions : 🔿 Yes 🔿 No

- - If yes : 🔿 every day 🔿 a few days a week

Did you suffer for anxiety lately : 🔿 yes 🔿 No

- - If yes your anxiety level was: 🔿 Normal 🔿 Increased 🔿 Very increased

Your emotional difficulties during restrictions are:

- The same 🔿 Increased 🔿 Decreased

Did you feel:

Boredom 🔿 Yes 🔿 No Dissatisfaction 🔿 Yes 🔿 No Fear 🔿 Yes 🔿 No

Depression 🔿 Yes 🔿 No Rage 🔿 Yes 🔿 No

Any difference about sleep quality /quantity? 🔿 Sì 🔿 No

- If yes (multiple response): ❑ Insomnia ❑ Early awakening ❑ Daytime nap

Have your lifestyle habits changed? 🔿 No 🔿 A little 🔿 Very much

Has your psycho-physical wellbeing changed? : 🔿 No 🔿 Incresead 🔿 Decreased

Are you dissatisfied with your body?: 🔿 Yes 🔿 No

- If yes: 🔿 as before 🔿 less than before 🔿 more than before

During the day , are you thinking about food: 🔿 Rarely 🔿 Only when I’m eating 🔿 Often 🔿 Continuously

Time thinking about food is: 🔿 Same of before 🔿 Decreased 🔿 increased

Has quarantine changed your perceived value of food? 🔿 Yes 🔿 No

- If Yes the value is: 🔿 Less than before 🔿 More than before

Thinking about food is for you: 🔿 Pleasure 🔿 Obsession 🔿 Fault

During restrictions for pandemic your hunger perception is:

- - Increased 🔿 Decreased 🔿 Unchanged 🔿 I’m not hungry but I need to eat

During restrictions you have eaten: 🔿 As before 🔿 More 🔿 Less

How did the restrictions related to the coronavirus pandemic impact your eating habits:

|  | No | Yes, unchanged | Yes, more than before | Yes, less than before |
| --- | --- | --- | --- | --- |
| I have regular meals |  |  |  |  |
| Snack between meals |  |  |  |  |
| I eat during the night |  |  |  |  |
| I eat calorie-dense food |  |  |  |  |
| I eat healthy and lower-calorie foods |  |  |  |  |
| I eat elaborate, high-fat meals |  |  |  |  |
| I am economically able to have balanced meals |  |  |  |  |
| I eat uncontrollably |  |  |  |  |

Did you cooked your meals during pandemic? 🔿 Yes 🔿 No 🔿 Sometimes

Did you prefer to prepare food at home that you previously bought ready-made? 🔿 Yes 🔿 No

How did the restrictions related to the coronavirus pandemic impact your shopping behaviour and consumption of the following foods:

| FOOD | Increased | Decreased | Unchanged |
| --- | --- | --- | --- |
| Legumes |  |  |  |
| Flour |  |  |  |
| Pasta |  |  |  |
| Bread |  |  |  |
| Food with wholemeal flours |  |  |  |
| Fresh fruit |  |  |  |
| Fresh vegetables |  |  |  |
| Sweets and salted snacks |  |  |  |
| Homemade sweets |  |  |  |
| Fish |  |  |  |
| Meat and sausages |  |  |  |
| Milk and yogurt |  |  |  |
| Cheese |  |  |  |
| Eggs |  |  |  |
| Coffee and tea |  |  |  |
| Wine, beer, spirits |  |  |  |
| Soft drinks |  |  |  |
| Olive oil |  |  |  |
| Dried fruit |  |  |  |
| Sugar |  |  |  |
| Sweeteners |  |  |  |

The restrictions due to the pandemic changed my physical activity (choose one):

- - I had to reduce my physical activity level compared to before the pandemic
  - I was able to replace outdoor activity with physical activity at home (eg use of gymnastics tutorials at home, exercise bike, treadmill) or in any case my physical activity did not significantly change
  - I have increased physical activity compared to before the pandemic

Since the start of the restrictions imposed by the pandemic, your weight is:

- - Unchanged 🔿 Reduced (-____ kg ) 🔿 Increased (+____kg)

Were you on diet therapy before pandemic? 🔿 Yes 🔿 No

Did you have difficulty monitoring it? 🔿 Yes 🔿 No

Did you take drugs for obesity before pandemic? 🔿 Yes 🔿 No

Would you have considered it appropriate to use obesity drugs in this period? 🔿 Yes 🔿 No

Were you able to keep in touch with your center / professional: 🔿 Yes 🔿 No

- If yes how?. ❑ Phone ❑ Email ❑ WhatsApp ❑ Skype/Videocall

The importance of the obesity disease for you in this period is:

🔿 Unchanged 🔿 Decreased 🔿 increased

Have you had bariatric surgery? 🔿 Yes 🔿 No

- If yes, when was your surgery? 🔿 Less than one year ago 🔿 More than one year ago
