# Supplementary material for: Determinants of weight, psychological status, food contemplation and lifestyle changes in patients with obesity during the COVID-19 lockdown: a nationwide survey using multiple correspondence analysis
Source: Int J Obes (Lond). 2022 Mar 19;46(7):1280–7. doi: 10.1038/s41366-022-01100-8 (PMC8933751; doi:10.1038/s41366-022-01100-8)
Supplement: Supplementary file 2 — Original Survey Questionnaire (Italian) [file 41366_2022_1100_MOESM2_ESM.pdf]

Fondazione ADI
QUESTIONARIO OBESITY DAY 2020

Il tuo stile di vita domiciliare ai tempi delle restrizioni da epidemia COVID-19

Queste domande hanno lo scopo di capire l'impatto delle restrizioni imposte dalla epidemia da Coronavirus sul tuo stile di vita

Data della compilazione __________
Centro Obesity Day ________________________________ **Città** ___________________________ **PR** ___

Sesso ❍ M ❍ F **Età** ____ **Peso** _____**kg.** **Altezza** _____**cm** **BMI** _______

Professione:
❍ Disoccupato ❍ Casalinga ❍ Artigiano/commerciante/agricoltore ❍ Dipendente pubblico
❍ Dipendente privato ❍ Libero professionista ❍ Pensionato ❍ Altro……….

Scolarità: ❍ Elementare ❍ Media Inferiore ❍ Media superiore ❍ Laurea

Lavoro agile (Smart working) in questi mesi: ❍ Sì ❍ No

Hai sospeso il lavoro: ❍ Sì ❍ No
-se sì per: ❍ Scelta personale ❍ Obbligata dalle restrizioni ❍ Cassa integrazione

Hai avuto la possibilità di uscire da casa : ❍ Sì ❍ No
- se Sì : ❍ Ogni giorno ❍ Qualche giorno la settimana

Hai sofferto di ansia in questi giorni : ❍ Sì ❍ No
- se Sì hai avuto un livello di ansia: ❍ Normale ❍ Aumentato ❍ Molto aumentato

Le tue difficoltà emotive precedenti all'isolamento in questo periodo sono:
❍ Le stesse ❍ Aumentate ❍ Diminuite

Hai provato:
Noia ❍ Sì ❍ No **Insoddisfazione** ❍ Sì ❍ No **Paure** ❍ Sì ❍ No
Depressione ❍ Sì ❍ No **Rabbia** ❍ Sì ❍ No

Hai riscontrato modifiche riguardo alla qualità /quantità del sonno? ❍ Sì ❍ No
- se Sì (risposta multipla): ❑ Insonnia ❑ Risveglio precoce ❑ Sonnellino diurno

Sono cambiate le tue abitudini di vita? ❍ Per niente ❍ Poco ❍ Molto

La percezione del tuo benessere psico-fisico è: ❍ Invariato ❍ Aumentato ❍ Diminuito

Ti senti insoddisfatto del tuo corpo: ❍ Sì ❍ No
- se Sì: ❍ Come prima ❍ Meno di prima ❍ Di più rispetto a prima

Nella tua giornata pensi al cibo: ❍ Raramente ❍ Solo quando mangio ❍ Spesso ❍ Continuamente

Il tempo che impieghi pensando al cibo rispetto a prima è: ❍ Lo stesso ❍ Diminuito ❍ Aumentato

La quarantena ha modificato il valore che ha per te il cibo ❍ Sì ❍ No
- se Sì è importante: ❍ Come prima ❍ Meno di prima ❍ Più di prima

In questo periodo il pensiero del cibo è per te: ❍ Piacere ❍ Ossessione ❍ Colpa

Da quando vi sono le restrizioni imposte dalla epidemia da Coronavirus la tua percezione di fame è:
❍ Aumentata ❍ Ridotta ❍ Invariata ❍ Non ho fame ma sento il bisogno di mangiare

In questo periodo hai mangiato rispetto a prima: ❍ Lo stesso ❍ Di più ❍ Di meno

Le restrizioni imposte dalla epidemia da Coronavirus hanno comportato le seguenti modifiche sulla tua alimentazione:

	No	Invariato	Più di prima	Meno di prima
Faccio pasti ad orari regolari				
Spilucco tra un pasto e l'altro				
Mangio la notte				
Mangio alimenti calorici				
Mangio alimenti sani e meno calorici				
Mangio pasti elaborati e ricchi in grassi				
Riesco economicamente ad effettuare pasti bilanciati				
Mangio in modo incontrollato				

Hai cucinato tu in questo periodo i pasti che hai consumato? ❍ Sì ❍ No ❍ A volte

Avete preferito preparare alimenti in casa che prima compravate già pronti? ❍ Sì ❍ No

Le restrizioni imposte dalla epidemia da Coronavirus hanno comportato modifiche sulla tua spesa/consumo dei seguenti alimenti:

Alimento	Aumentato	Ridotto	Invariato
Legumi			
Farina			
Pasta			
Pane			
Alimenti da farine integrali			
Frutta Fresca			
Verdura Fresca			
Dolciumi E Snack Salati			
Dolci fatti in casa			
Pesce			
Carni e insaccati			
Latte e yogurt			
Formaggi			
Uova			
Caffè E Thè			
Alcolici (Vino, Birra, Superalcolici)			
Bevande Dolci (Cole, Succhi di frutta ecc.)			
Olio di oliva			
Frutta secca			
Zucchero			
Dolcificanti			

Le restrizioni imposte dalla epidemia da Coronavirus hanno comportato le seguenti modifiche sulla tua attività motoria (scegliere una risposta):
❍ Ho dovuto ridurre l'attività motoria rispetto a prima.
❍ Sono riuscito a sostituire l'attività all'aperto con attività motoria in casa (es. utilizzo di tutorial di ginnastica a casa, cyclette, tapis roulant) o comunque la mia attività motoria non si è significativamente modificata.
❍ Ho aumentato l'attività motoria rispetto a prima

Da quando vi sono le restrizioni imposte dalla epidemia da Coronavirus il tuo peso è:
❍ Invariato ❍ Ridotto (-____ kg) ❍ Aumentato (+____kg)

Eri già in dietoterapia prima di questo periodo? ❍ Sì ❍ No

Hai avuto difficoltà nell'osservarla? ❍ Sì ❍ No

Hai utilizzato farmaci per l'obesità in questo periodo? ❍ Sì ❍ No

Avresti ritenuto opportuno utilizzare farmaci per l'obesità in questo periodo? ❍ Sì ❍ No

Sei riuscito a rimanere in contatto con il tuo centro/professionista: ❍ Sì ❍ No
- Se Sì come. Per: ❑ Telefono ❑ Email ❑ WhatsApp ❑ Skype/Videochiamata

L'importanza della malattia obesità per te in questo periodo è: ❍ Invariata ❍ Diminuita ❍ Aumentata

Sei stato operato di chirurgia bariatrica? ❍ Sì ❍ No
- Se Sì da quanto tempo? ❍ Meno di un anno ❍ Più di un anno
